# Effects of Preoperative Quadruple Therapy for *Helicobacter pylori* on Bariatric Surgery Metabolic Outcomes

**DOI:** 10.1007/s11695-024-07091-x

**Published:** 2024-02-24

**Authors:** Albert Goday, Andrea Bagán, Anna Casajoana, Carme Serra, Manuel Pera, Montserrat Villatoro, Teresa Legido, Helena Julià, Elisenda Climent, Olga Castañer, Juana A Flores Le Roux, Miguel Olano, Juan Pedro-Botet, David Benaiges

**Affiliations:** 1 Department of Endocrinology and Nutrition, Hospital del Mar, 08003 Barcelona, Spain; 2 Departament de Medicina, Universitat Autònoma de Barcelona, Barcelona, Spain; 3 Cardiovascular Risk and Nutrition Research Group (CARIN-ULEC), IMIM-Hospital del Mar, Barcelona. Biomedical Research Park (Parc de Recerca Biomèdica de Barcelona– PRBB), 08003 Barcelona, Spain; 4 Centro de Investigaciones Biomédicas en Red de Obesidad y Nutrición, CIBERobn, Madrid, Spain; 5 Department of Medicine, Universitat Pompeu Fabra. Plaça de la Mercè, 10-12, E-08002 Barcelona, Spain; 6 Unit of Gastrointestinal Surgery, Hospital del Mar, Institut de Recerca IMIM- Hospital del Mar, 08003 Barcelona, Spain; 7 Neuroscience Group, Hospital del Mar Medical Research Institute, 08003 Barcelona, Spain; 8 Department of Endocrinology and Nutrition, Althaia, Xarxa Assistencial Universitària de Manresa, Manresa, 08243 Barcelona, Spain; 9 Consorci Sanitari de l’Alt Penedès i Garraf, 08720 Vilafranca del Penedès, Spain

**Keywords:** *Helicobacter pylori*, Bariatric surgery, Morbid obesity, Gastric bypass, Sleeve gastrectomy

## Abstract

**Purpose:**

To assess the effects of *Helicobacter pylori* (HP) eradication with an omeprazole, clarithromycin, amoxicillin, and metronidazole (OCAM) regimen on the metabolic profile and weight loss 12 months after bariatric surgery (BS).

**Methods:**

Retrospective analysis of a prospective cohort of patients with morbid obesity undergoing BS. HP presence was tested preoperatively by gastric biopsy and treated with OCAM when positive. Short-term metabolic outcomes and weight loss were evaluated.

**Results:**

HP infection was detected in 75 (45.7%) of the 164 patients included. OCAM effectiveness was 90.1%. HP-negative patients had a greater reduction in glucose levels at 3 (−14.6 ± 27.5 mg/dL HP-treated vs −22.0 ± 37.1 mg/dL HP-negative, *p*=0.045) and 6 months (−13.7 ± 29.4 mg/dL HP-treated vs −26.4 ± 42.6 mg/dL HP-negative, *p*= 0.021) and greater total weight loss (%TWL) at 6 (28.7 ± 6.7% HP-treated vs 30.45 ± 6.48% HP-negative, *p*= 0.04) and 12 months (32.21 ± 8.11% HP-treated vs 35.14 ± 8.63% HP-negative, *p*= 0.023).

**Conclusions:**

Preoperative treatment with OCAM has been associated to poorer glycemic and weight loss outcomes after BS. More research is needed on the influence of OCAM on gut microbiota, and in turn, the effect of the latter on metabolic and weight loss outcomes after BS.

**Graphical Abstract:**

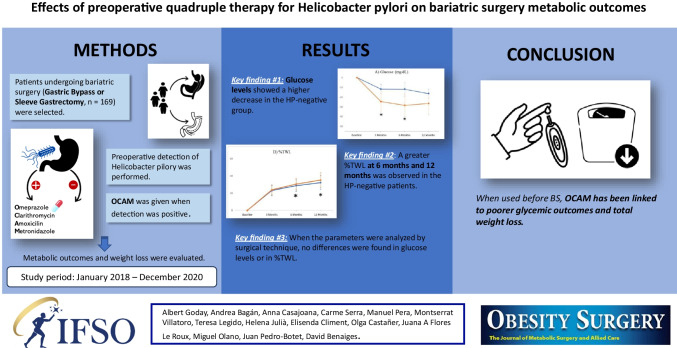

## Introduction

Bariatric surgery (BS) is a recognized long-term treatment for severe obesity [1, 2], with improvement in associated comorbidities such as type 2 diabetes, hypertension, dyslipidemia, and sleep apnea [3]. In recent years, comorbidity recovery has been found to be a complex issue in which not only weight loss is involved, and multiple mechanisms such as hormonal changes, bile acids, epigenetics, or modifications in gut microbiota as well as others are also related [4, 5].

On the other hand, *Helicobacter pylori* (HP) infection is common in patient candidates for BS. The current guidelines recommend its eradication before surgery, since this infection could potentially be associated with postoperative complications [6–8]. Previous studies suggested that HP infection and mainly its treatment with an omeprazole, clarithromycin, and amoxicillin (OCA) regimen prior to intervention could have a modulating effect on the metabolic response to BS [9].

It must be highlighted that metronidazole has been added to OCA as the standard first-line HP treatment in the last 3 years. Several studies comparing the effectiveness of this new combination, known as OCAM versus OCA, have consistently confirmed OCAM as the better option [10, 11]. Nevertheless, very few studies have tested OCAM’s effectiveness in patients with obesity undergoing BS [12] and none have assessed the effects of OCAM on metabolic outcomes following BS.

Taking into account all of the above, the main aim of the present study was to examine the effects of HP eradication using the OCAM regimen on the metabolic profile and weight loss 12 months after BS. The secondary aim was to assess the effectiveness of OCAM treatment on HP eradication in patients with clinically severe obesity candidates for BS.

## Materials and Methods

### Study Protocol

This study was a retrospective analysis of a prospective cohort of patients with morbid obesity who underwent BS between January 2018 and December 2020 at the Hospital del Mar in Barcelona, Spain. Patients aged between 18 and 60 years who met the 1991 National Institute of Health BS criteria were included [13]. Patients with any other condition that did not allow them to undergo BS and those who did not complete the 12 months of follow-up were excluded. Based on clinical criteria and the consensus of the BS unit, patients were assigned to gastric bypass (GB) or sleeve gastrectomy (SG). All patients signed their informed consent for the procedure and for the study.

Patients were evaluated by a multidisciplinary team preoperatively and at 3, 6, and 12 months after surgery. During the preoperative period, group sessions were performed under the supervision of a registered dietitian, in order to conduct a structured dietary intervention which consisted in two parts. In the first part, patients were instructed with nutritional advice to encourage a 5–10% body weight loss prior to surgery. In the second part, patients followed a very low-calorie diet 2 weeks prior surgery. In the early postoperative period, patients were trained on a progressive diet adaptation. The visit protocol included anthropometric and blood pressure measurements, as well as glucose, insulin, glycated hemoglobin (HbA1C), total cholesterol, high-density lipoprotein (HDL) cholesterol, low-density lipoprotein (LDL) cholesterol, and triglyceride levels. Weight loss was reported as percentage of total weight loss (%TWL). Preoperative %EWL refers to the weight loss that occurs during the preoperative phase of the treatment, which encompasses the period from when patients are referred to our unit until they undergo the surgical procedure.

Glucose was determined by the oxidase method. HbA1C was quantified by chromatography. Insulin was measured by radioimmunoassay. The HOMA-IR index was estimated using the following formula: insulin (mU/mL) × fasting glucose (mmol/L)/22.5 [14]. Total cholesterol and triglyceride concentrations were determined using enzymatic methods by a Cobas Mira automatic analyzer (Baxter Diagnosis AG, Dündingen, Switzerland). HDL cholesterol was measured using separation by precipitation with phosphotungstic acid and magnesium chloride. LDL cholesterol concentration was calculated by the Friedewald formula.

### *Helicobacter pylori* Detection and Treatment

All patients underwent upper-gastrointestinal endoscopy preoperatively to rule out esophageal, gastric, and/or duodenal lesions. Histologic analysis was then used to assess the presence or absence of HP. If this was not possible, a breath test with 13C-urea was done. The concordance between tests has previously been reported [15–17]. If HP was present, patients received eradication treatment. In accordance with Maastricht V recommendations, the first-line treatment was 14 days of quadruple therapy: proton pump inhibitor omeprazole (20 mg twice a day), clarithromycin (500 mg twice a day), amoxicillin (1 g twice a day), and metronidazole (500 mg) twice a day. Effectiveness was assessed by a breath urease test [18] 2 months after treatment. In the case of therapeutic failure, a second-line treatment was prescribed for 14 days (omeprazole 40 mg twice a day, amoxicillin 1 g twice a day and levofloxacin 500 mg twice a day or bismuth subcitrate 140 mg four times a day, tetracycline 125 mg four times a day and metronidazole 125 mg four times a day). All surgical specimens were analyzed for HP presence, fundus gastrectomy in SG, and anastomotic rings in GB.

### Surgical Techniques

The GB technique involved a 150-cm antecolic Roux limb with 25-mm circular pouch-jejunostomy and 50-cm proximal jejunum exclusion. In SG, a longitudinal stomach resection, from 5 cm proximal to the pylorus to the His angle was performed using a 38-French bougie inserted along the lesser curvature [19].

### Statistical Analysis

All patients with HP presence and who received treatment were considered for analysis as HP-treated. Those who tested negative and subsequently did not receive treatment were considered HP-negative. The outcomes were analyzed and presented for all patients in general, as well as separated by the type of surgical technique.

Data were expressed as mean ± standard deviation for continuous variables and as percentages and frequencies for categorical variables. Variables that did not follow a normal distribution were log transformed for analysis to achieve normality. Student’s *t*-test for independent samples was used to compare variables according to HP status at each time point. ANOVA test for repeated measurements was used to assess changes during follow-up. Chi-square test was used to compare proportions between groups. *p* values less than 0.05 were considered statistically significant. Linear regression analysis was applied to evaluate factors independently associated with weight loss. All variables associated with weight loss on univariate analysis (*p*< 0.1) were included in the regression model. Data were analyzed with the statistical software package IBM SPSS Statistics V.25.0.

## Results

**Fig. 1:**
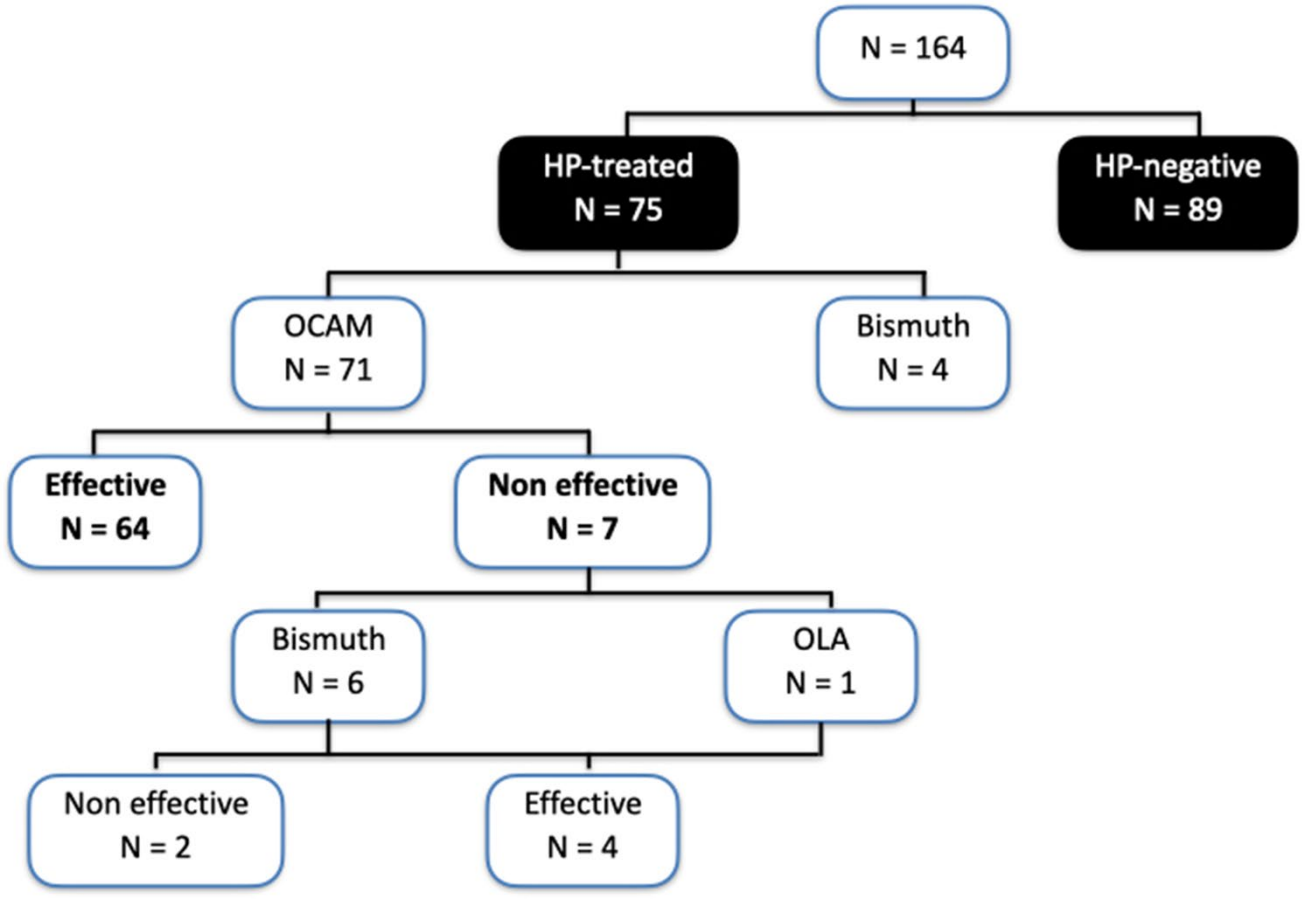
*Helicobacter pylori* treatment flowchart diagram. HP, *Helicobacter pylori*; OCAM, omeprazole, clarithromycin, amoxicillin, metronidazole; OLA, omeprazole, levofloxacin, amoxicillin; Bismuth, quadruple therapy with bismuth subcitrate, tetracycline, metronidazole

Nine (5%) of the 173 patients who underwent BS were excluded owing to lack of follow-up beyond 12 months. Out of the nine patients excluded due to loss of follow-up, 5 were HP-negative, and 4 were HP-positive. Of the 164 included patients, 96 underwent SG and 68 GB. The flowchart of HP status and treatment is shown in Fig. 1; 75 patients (45.7%) were HP-positive and OCAM was effective in 64 patients (90.1%). All surgical samples were analyzed for HP presence, with 152 being negative and 12 positive. Time from the start of eradication treatment to BS was 10.0 ± 4.7 months.

The clinical characteristics of patients according to HP status and surgical technique are shown in Table 1. The only difference found was that HP-negative patients underwent GB in a significantly greater proportion than HP-treated patients (*p*= 0.037). Within each of the surgical procedures, no differences were observed in baseline clinical parameters except for triglyceride concentrations, which were lower in the HP-treated group of the patients who underwent GB (*p*= 0.041) (Table 2).

**Table 1:** Baseline characteristics of severely obese patients undergoing bariatric surgery according to *Helicobacter pylori* infection status

	HP-negative (*n*=89)	HP-treated (*n*=75)	*p* value
Age (years)	46.7 ± 9.7	47.5 ± 9.5	0.577
Female (%)	77.5	76.0	0.481
BMI (kg/m^2^)	44.0 ± 4.3	44.4 ± 6.5	0.644
Hypertension (%)	37.9	41.1	0.403
Systolic blood pressure (mmHg)	141.5 ± 17.6	139.0 ± 21.7	0.427
Diastolic blood pressure (mmHg)	85.4 ± 10.6	84.4 ± 9.8	0.537
Glycemia (mg/dL)	114.0 ± 36.6	110.5 ± 34.7	0.567
Insulin (mU/mL)	17.5 ± 30.6	19.9 ± 24.9	0.291
HOMA-IR	5.0 ± 3.5	5.7 ± 5.2	0.335
HbA1C (%)	5.7 ± 1.1	5.7 ± 0.9	0.777
Total cholesterol (mg/dL)	189.1 ± 35.4	186.3 ± 34.0	0.608
HDL cholesterol (mg/dL)	51.4 ± 10.5	56.4 ± 14.9	0.394
LDL cholesterol (mg/dL)	114.6 ± 25.8	109 ± 27.0	0.697
Triglycerides (mg/dL)	133.1 ± 62.5	139.5 ± 114.5	0.67
Diabetes (%)	25.0	23.3	0.474
Dyslipidemia (%)	29.5	20.5	0.131
Cigarette smoking (%)	15.9	20.5	0.288
GB (%)	48.3	33.3	0.037

*HP Helicobacter pylor*i, *BMI* body mass index, *HOMA-IR* insulin resistance, *HbA1C* glycated hemoglobin, *HDL* high-density lipoprotein, *LDL* low-density lipoprotein, *GB*, gastric bypass

**Table 2:** Baseline characteristics of severely obese patients undergoing bariatric surgery according to initial *Helicobacter pylori* infection status and separated by surgical technique

	Gastric bypass			Sleeve gastrectomy		
	HP-negative (*n*=43)	HP-treated (*n*=25)	*p* value	HP-negative (*n*=46)	HP-treated (*n*=50)	*p* value
Age (years)	46.6±9	45.4±9.8	0.590	46.7±10.4	48.6±9.3	0.35
Female (%)	86.0	84.0	0.54	69.6	72.0	0.485
BMI (kg/m^2^)	45.2±4.7	45.0±4.7	0.824	42.8±3.5	44.1±7.3	0.285
Hypertension (%)	31.7	50.0	0.116	43.5	36.7	0.322
Systolic blood pressure (mmHg)	141.4±16,2	143.6±16.4	0.595	141.6±19.0	136.8±23.8	0.28
Diastolic blood pressure (mmHg)	85.4±10.8	84.0±10.7	0.635	85.4±10.6	84.5±9.5	0.676
Glycaemia (mg/dL)	118.2±48.1	114.1±34.7	0.712	110.2±33.0	108.8±35.6	0.841
Insulin (mU/ml)	17.3±10.5	19.3±8.8	0.466	17.6±11.4	20.2±19.9	0.449
HOMA-IR	5.1±3.4	5.6±3.9	0.634	4.9±3.4	5.8±6.1	0.415
HbA1C (%)	5.9±1.3	5.6±0.9	0.350	5.6±0.9	5.7±1.0	0.492
Total cholesterol (mg/dL)	194.9±37	182.6±27.9	0.154	183.4±35.6	188.2±32.5	0.503
HDL cholesterol (mg/dL)	51.1±9.6	48.0±11.5	0.239	51.7±11.9	60.7±64.3	0.36
LDL cholesterol (mg/dL)	116.2±30	100.6±15.2	0.381	112.1±22.8	125.7±45.7	0.676
Triglycerides (mg/dL)	143.5±76.5	108.9±40.7	0.041	123.6±54.0	155.2±143.4	0.169
Diabetes (%)	33.3	29.2	0.474	17.4	20.4	0.456
Dyslipidemia (%)	33.3	16.7	0.119	26.1	22.4	0.431
Cigarette smoking (%)	16.7	12.5	0.736	15.2	24.5	0.311

*HP Helicobacter pylori*, *BMI* body mass index, *HOMA-IR* insulin resistance, *HbA1C* glycated hemoglobin, *HDL* high-density lipoprotein, *LDL* low-density lipoprotein, *GB* gastric bypass

**Fig. 2:**
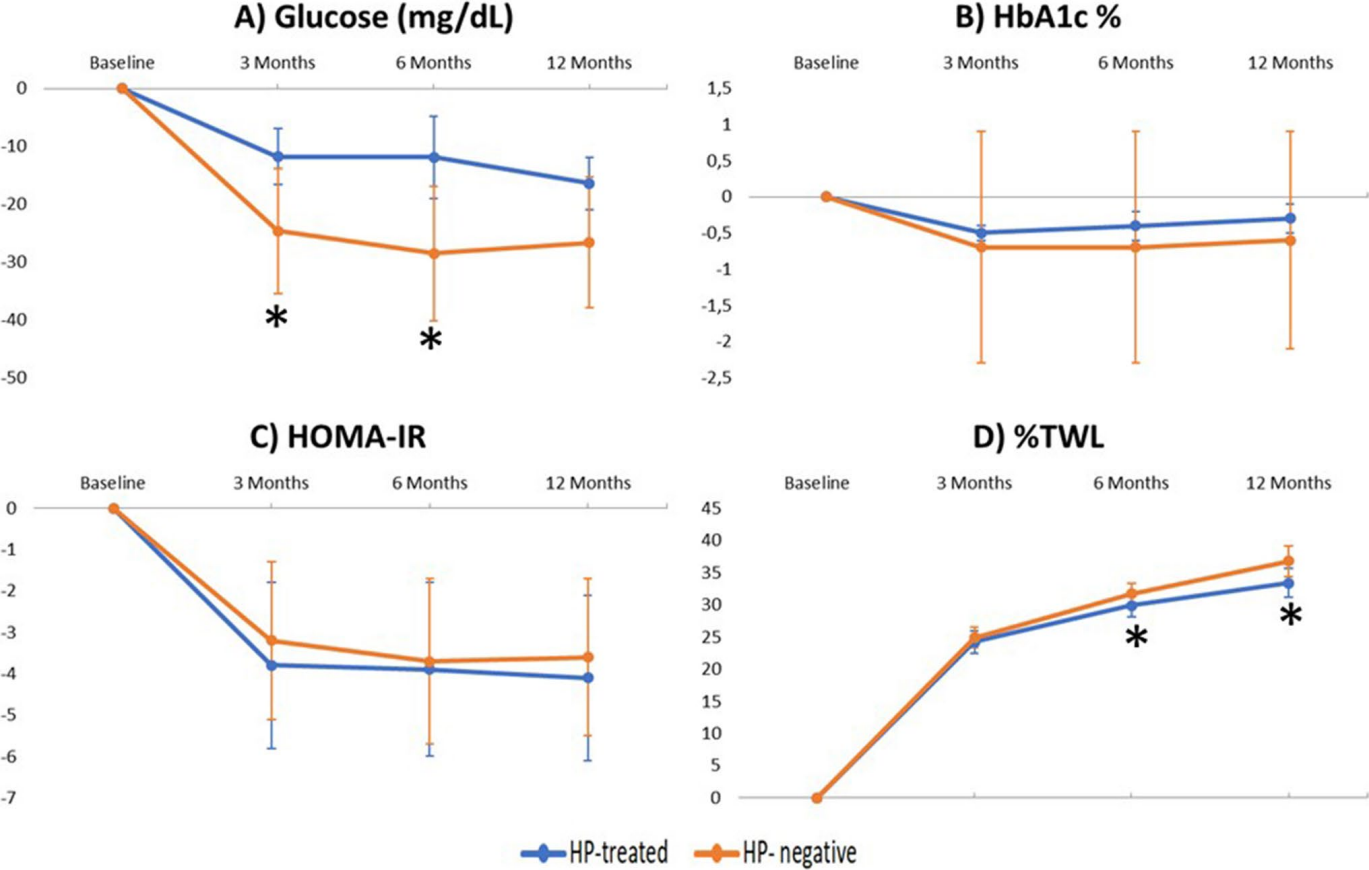
Evolution of **A** glucose, **B** HbA1c, **C** HOMA-IR, and **D** percentage total weight loss (%TWL) after bariatric surgery according to initial *Helicobacter pylori* status. Data are expressed as means (95% confidence interval). Negative values indicate a reduction while positive values indicate increase. *p< 0.05

**Fig. 3:**
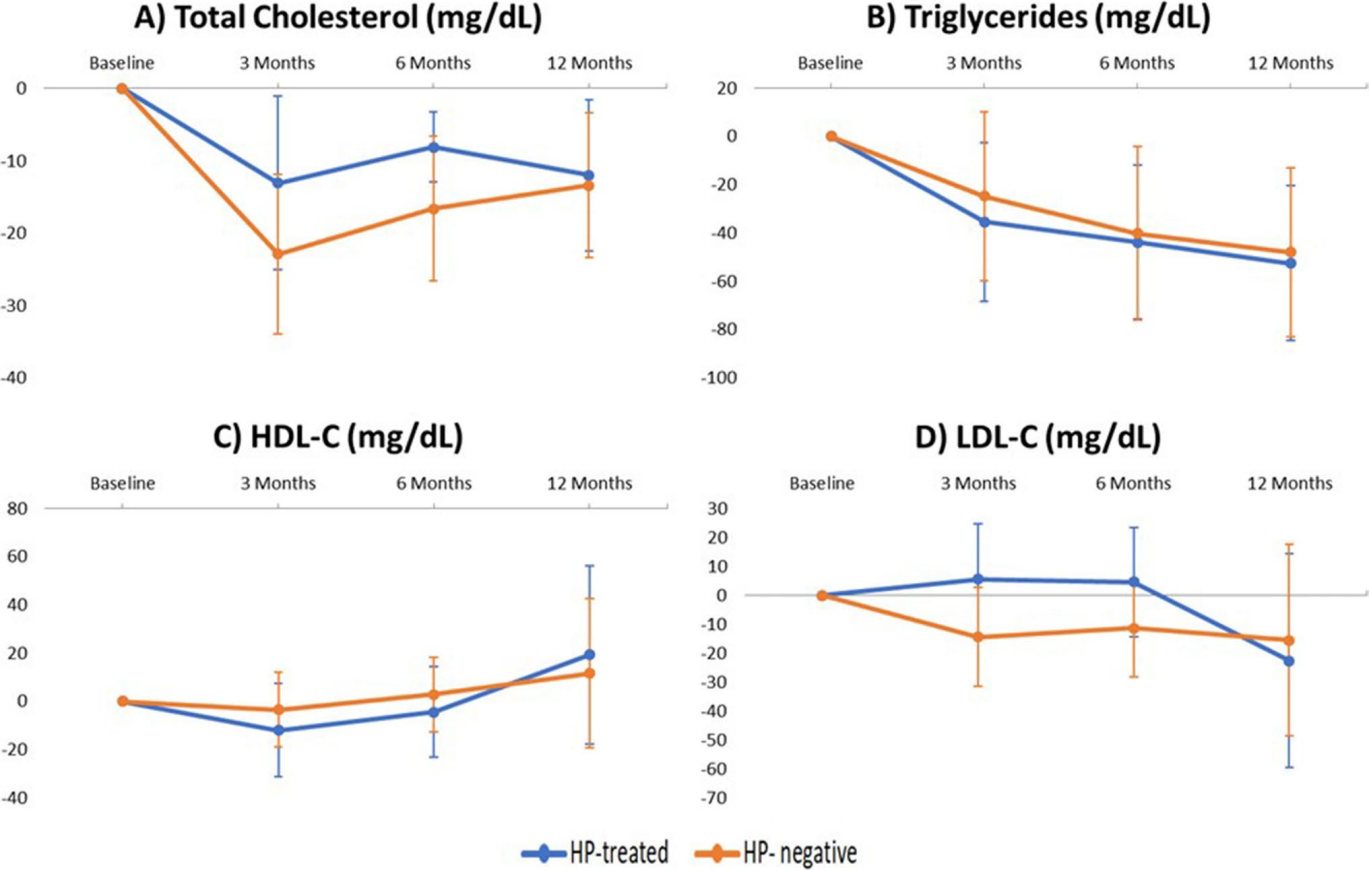
Evolution of **A** total cholesterol, **B** triglycerides, **C** HDL cholesterol, and **D** LDL cholesterol after bariatric surgery according to initial *Helicobacter pylori* status. Data are expressed as means (95% confidence interval). Negative values indicate a reduction while positive values indicate an increase. *p< 0.05

**Fig. 4:**
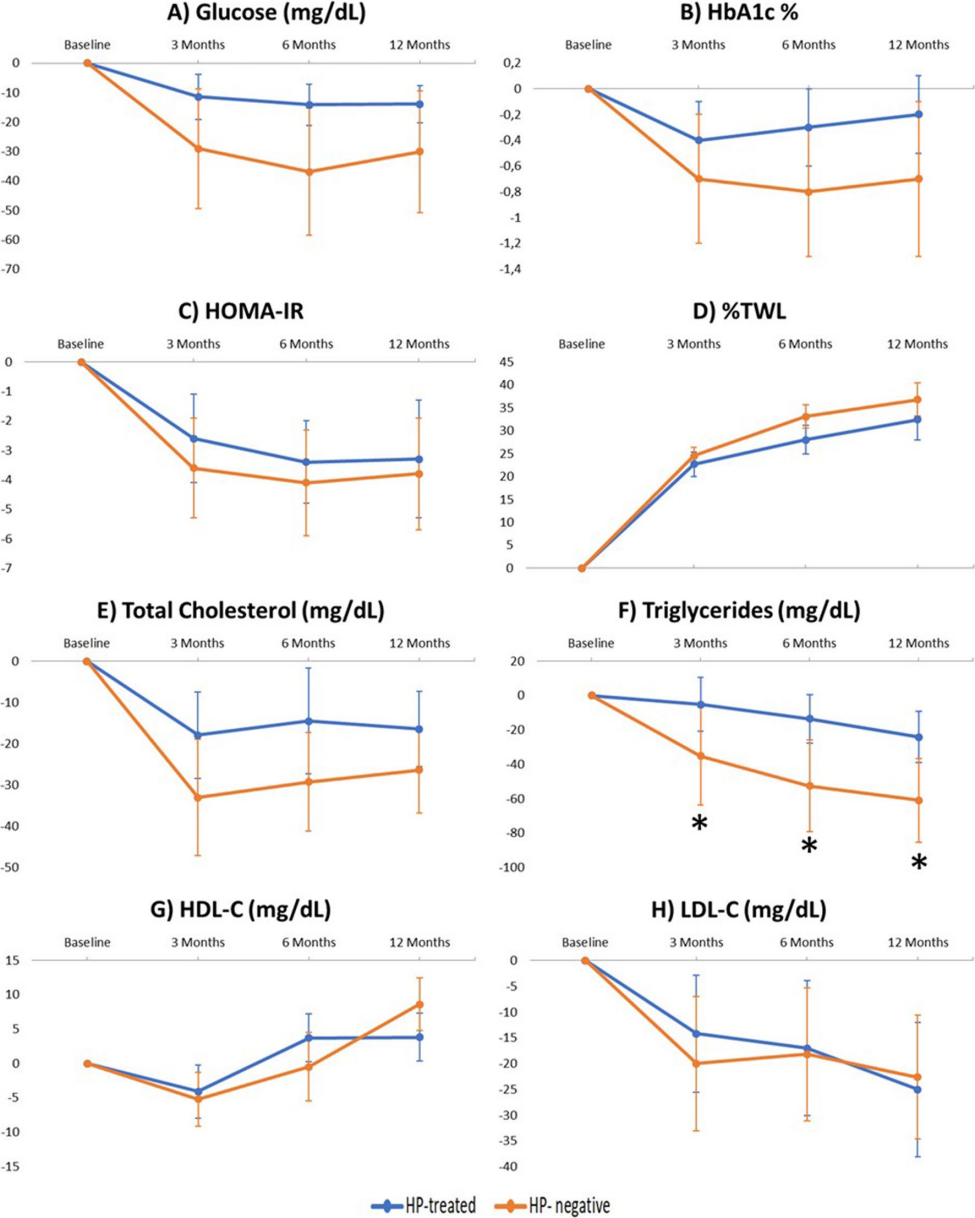
Evolution of **A** glucose, **B** HbA1c, **C** HOMA-IR, **D** percentage total weight loss (%TWL), **E** total cholesterol, **F** triglycerides, **G** HDL cholesterol, and **H**) LDL cholesterol after gastric bypass according to initial *Helicobacter pylori* status. Data are expressed as means (95% confidence interval). Negative values indicate a reduction while positive values indicate an increase. *p< 0.05

**Fig. 5:**
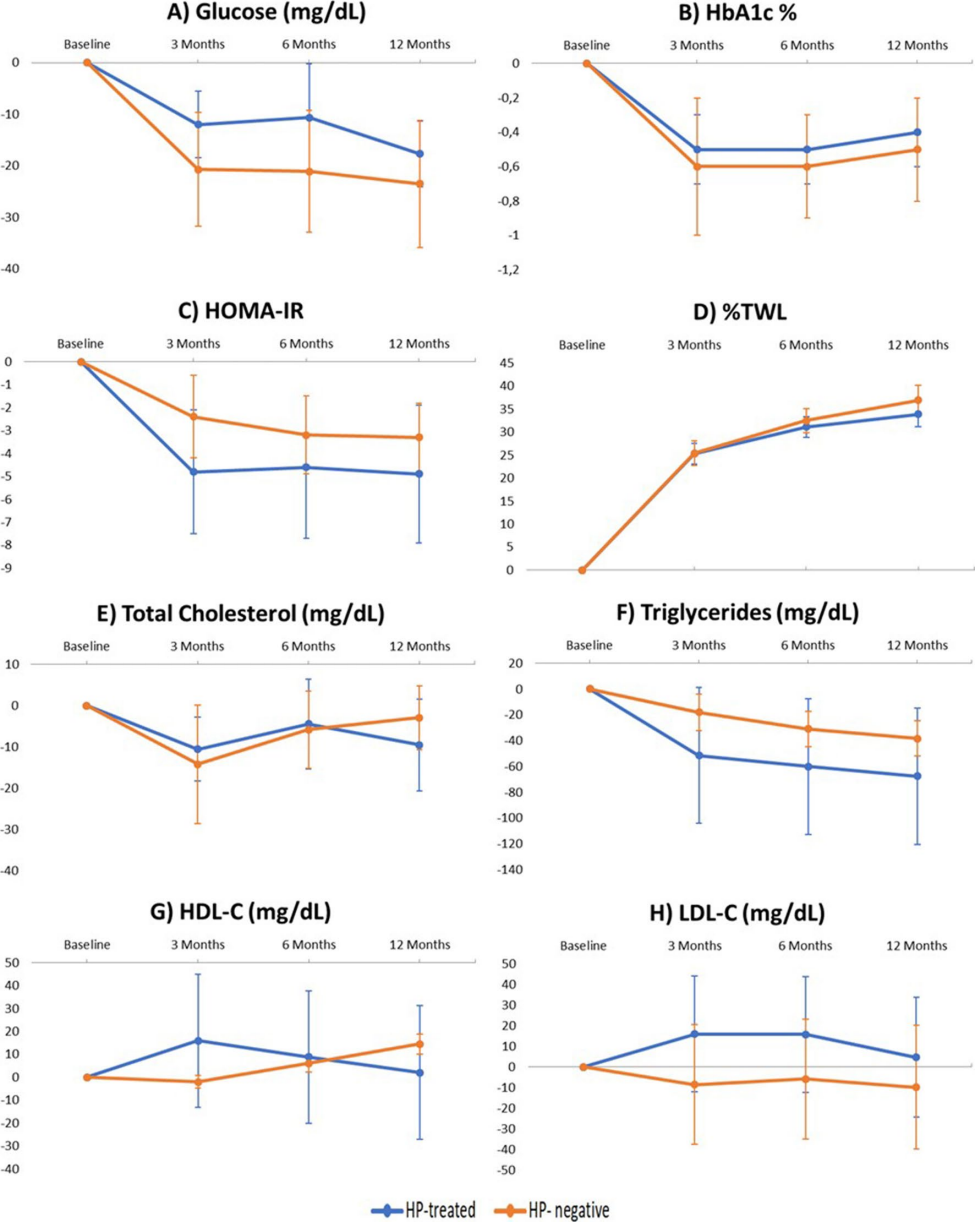
Evolution of **A** glucose, **B** HbA1c, **C** HOMA-IR, **D** percentage total weight loss (%TWL), **E** total cholesterol, **F** triglycerides, **G** HDL cholesterol, and **H** LDL cholesterol after sleeve gastrectomy according to initial *Helicobacter pylori* status. Data are expressed as means (95% confidence interval). Negative values indicate a reduction while positive values indicate an increase. *p< 0.05

HbA1c, total cholesterol, LDL, HDL, and triglyceride levels and HOMA-IR showed no significant differences between the HP-treated and HP-negative groups during the 12-month follow-up period, as shown in Figs. 2 and 3. With regard to glucose levels, significant differences in the HP-treated group were found compared to the HP-negative group at 3 months (−14.6 ± 27.5 mg/dL HP-treated vs −22.0 ± 37.1 mg/dL HP-negative, *p*= 0.045) and at 6 months (−13.7 ± 29.4 mg/dL HP-treated vs −26.4 ± 42.6 mg/dL HP-negative, *p*= 0.021) (Fig. 2). The results showed that the decline in glucose levels was greater in the HP-negative group. Moreover, the HP-negative group had a greater %TWL at 6 months (28.7 ± 6.7% HP-treated vs 30.45 ± 6.48% HP-, *p*= 0.04) and 12 months (32.21 ± 8.11% HP-treated vs 35.14 ± 8.63% HP-negative, *p*= 0.023) than HP-treated patients (Fig. 2). When the parameters were analyzed by surgical technique, no differences were found in glucose levels or in %TWL. However, a greater decrease in triglycerides was observed in the HP-negative patients who underwent GB (Figs. 4 and 5).

To deeply study the relationship between weight loss and HP treatment, a multivariate analysis was performed. Age (*p*= 0.02), HP-treated (*p*= 0.034), and preoperative %EWL (*p* < 0.001) were independently associated with 12-month %TWL (Table 3).

**Table 3:** Factors related to 12 months percentage total weight loss

Variables	*β* (95% CI)	*p* value	R^2^
Constant	94.5 [74.8–114.2]	<0.001	0.256
HP-treated	−7.1 [(−13.7)–(−0.6)]	0.034	
Sex	3.8 [(−3.9)–11.4]	0.334	
Age	−0.5 [(−0.8)–(−0.1)]	0.02	
Surgical technique	2 [(−4.8)–8.9]	0.561	
Diabetes	−4.1 [(−12.5)–4.4]	0.34	
Smoking	7 [(−2.1)–16.1]	0.129	
Hypertension	−1.7 [(−8.9)–5.7]	0.655	
Preoperative loss of excess weight	0.5 [0.3–0.8]	<0.001	

*HP Helicobacter pylori*

## Discussion

The present study is pioneer in assessing the effects of HP eradication with OCAM on the metabolic outcomes of patients with obesity undergoing BS. After surgery, a different glucose and weight loss evolution was noted between HP-negative and HP-treated patients. Moreover, the association between weight loss and HP status was found to be independent of the surgical technique and other factors.

Regarding the postoperative follow-up, a worse evolution of blood glucose and less weight loss was observed in subjects treated with OCAM compared with those that were not treated. These results contrast with those observed in a previous study by our center using OCA as HP treatment in which the best metabolic outcomes for glucose and triglyceride levels were found in the HP-treated group rather than the HP-negative group, and different results were found depending on the surgical technique.

Therefore, both studies suggest that preoperative antibiotic therapy could play a role regarding the evolution of some metabolic factors. In the OCA study, it was suggested that the observed results could be explained, at least in part, due to effect of antibiotics on gut microbiota. This has been a prominent research area in recent years, thus evaluating the link between human metabolism, intestinal epithelium homeostasis, and insulin resistance [20]. Following this line, it is known that some bacterial phyla have a beneficial effect on metabolic homeostasis, while others have a negative impact. The main difference between OCA and OCAM is the addition of metronidazole; this antibiotic targets gram-positive, gram-negative, and anaerobic bacteria, thus potentially causing completely different changes in gut microbiota, which could explain the present findings. This hypothesis is supported by two previous studies. In this sense, Jacobson et al. [21] reported that patients who received levofloxacin with metronidazole as antibiotic prophylaxis prior to SG lost less weight than those who received cefoxitin. Furthermore, Rodrigues et al. [22], in an animal model, found that glucose tolerance improved with different antibiotic regimens, except metronidazole.

Two main mechanisms could explain the relationship between microbiota and the regulation of body weight and metabolic changes. Firstly, through the modification of the caloric extraction capacity of food [23], and secondly, through its connection to the regulation of systemic inflammatory processes, which could be related to the development of obesity [24]. Therefore, we hypothesize that by modifying the microbiota through OCAM, these mechanisms would be modulated, and the observed changes could be explained.

A further factor that could be involved in the metabolic changes after BS is HP infection itself. In this respect, Gutierrez-Repiso et al. [25] in a cohort of 41 patients who had not received antibiotics in the previous months reported that those in whom HP infection was detected in the surgical sample after SG had less diversity in gastric microbiota and higher glucose levels, together with a more discrete body weight/BMI reduction 1 year after surgery. In the present study, this was probably not the predominant mechanism since the HP infection was treated several months before surgery, and therefore the gastric microbiota would have been expected to have regenerated.

With respect to %TWL, the differences observed between groups cannot be justified by the fact that a greater proportion of HP-negative patients were submitted to GB. Accordingly, the superiority of GB on weight loss has been demonstrated in mid- and long-term follow-up (from 3 years after surgery), but not 1 year after surgery [26]. Moreover, the multivariate analysis concluded that the surgical technique was not an independent factor contributing to %TWL. Instead, age, preoperative %TWL, and HP presence were found to be independent factors. In this sense, both age and preoperative excess weight loss have been previously recognized as associated factors in previous studies [27, 28]. Regarding the observed greater decrease in triglyceride levels in HP-negative patients within the GB cohort, it is likely to be related to the higher preoperative levels in this specific group.

Some bacterial and viral infections’ prevalence increase over time. Regarding the time period evaluated in the present study (2018–2020), HP infection prevalence in severely obese patients undergoing BS was 46%. A previous study of our group, in this case conducted between 2010 and 2013, observed a similar HP infection prevalence (48%) [9]. Therefore, no differences in HP infection rate in the last 10 years were detected in our Mediterranean area.

In the OCA study, the eradication effectiveness in patients with obesity undergoing BS was 78%, which was consistent with evidence at the time (2010–2013). By contrast, OCAM’s effectiveness in the present study was 90%. It should be emphasized that these are patients with morbid obesity in whom the pharmacokinetics of the drugs might have been altered, and consequently the standard dosage might have been insufficient. Fortunately, that was not the case. The conventional dosage was effective in a high percentage of patients, and was similar to the effectiveness described in other series of patients without severe obesity [29–31]. Only two other studies have assessed OCAM’s effectiveness in patients with obesity undergoing BS, and reported lower effectiveness rates (69.3% in 2012 and 71.6% in 2016) [11, 32]. These more modest results do not seem to be related to a worse pharmacokinetic behavior, but more to the fact that they were performed in a population with known resistances of HP to clarithromycin and metronidazole.

This study presents certain limitations. The main is the lack of a gut microbiota analysis before and after HP eradication. Therefore, it is not possible to know the impact of antibiotic therapy, rendering it difficult to pinpoint the exact cause of the study outcomes. As previously mentioned, the study with the OCA regime obtained different metabolic outcomes depending on the surgical technique (GB and SG). These results were not observed in the present study (except for triglyceride levels in patients submitted to GB), possibly due to the lack of statistical power to detect differences between both surgical groups. No factors that may have played a role in %TWL, such as physical activity, eating pattern, or quality of life, were considered. Socioeconomic status was not analyzed as a potential confounding variable.

## Conclusions

The present findings reinforce the choice of OCAM instead of OCA as a first-line HP infection treatment. However, when used before BS, OCAM has been linked to poorer glycemic outcomes and total weight loss. Thus, more research on the OCAM impact on gut microbiota is needed, in order to determine its specific effect on metabolic and weight loss outcomes after BS.

## Declarations

### Board Approval

The study was conducted in accordance with the Declaration of Helsinki, and approved by an Ethics Committee (2017/7722). “All procedures performed in studies involving human participants were in accordance with the ethical standards of the institutional and/or national research committee and with the 1964 Helsinki declaration and its later amendments or comparable ethical standards.”
